# Comparing the effects of text messaging and mobile social networking on physical activity and anthropometric indices of middle-aged women: a randomized controlled trial

**DOI:** 10.1186/s12905-022-01598-0

**Published:** 2022-01-26

**Authors:** Kousar Ansari, Poorandokht Afshari, Parvin Abedi, Mohammadhossein Haghighizadeh

**Affiliations:** 1 Midwifery Department, Reproductive Health Promotion Research Center, Ahvaz Jundishapur University of Medical Sciences, Ahvaz, Iran; 2 Midwifery Department, Reproductive Health Promotion Research Center, Nursing and Midwifery School, Ahvaz Jundishapur University of Medical Sciences, Golestan Ave, Ahvaz, Iran; 3 Midwifery Department, Menopause Andropause Research Center, Ahvaz Jundishapur University of Medical Sciences, Ahvaz, Iran; 4 Faculty of Public Health, Department of Statistics and Epidemiology, Ahvaz Jundishapur University of Medical Sciences, Ahvaz, Iran

**Keywords:** Social networking, Mobile, Text messaging, Anthropometric indices, Middle-aged women

## Abstract

**Background:**

Physical inactivity is a global health problem which is more prevalent among women. Among different age groups, middle-aged women are more vulnerable to physical inactivity as one of consequences of menopause. This study aimed to compare the effect of text messaging and that of mobile social networking on the improvement of physical activity and anthropometric indices of middle-aged women in Iran.

**Methods:**

This was a randomized controlled trial in which 110 overweight or obese women who were physically inactive were recruited and allocated into two groups of text messaging (n = 55) or mobile social networking (n = 55). Women in both groups received information regarding the necessity, benefits, and barriers of physical activity and how to overcome these barriers for 12 weeks. The International Physical Activity Questionnaire (IPAQ), and a demographic questionnaire were used to collect data. Anthropometric indices including weight, height, waist circumference, hip circumference, waist/hip ratio, and body fat percentage were measured at baseline, as well as 4, 8, and 12 weeks after intervention. Data were analyzed using independent *t*-test, Chi-square, and repeated measure test.

**Results:**

In the mobile social networking group, most women had moderate physical activity after 12 weeks (*P* < 0.05). The mean physical activity and energy expenditure were significantly higher in the mobile social group than those in the text messaging. In the 12th week of intervention, there was a significant reduction in the weight and BMI of the participants in the mobile social networking group compared to the text messaging group (*P* < 0.05). The waist and hip circumferences of women in the mobile social networking group reduced significantly after 12 weeks of intervention in comparison to the text messaging group (*P* = 0.001). The two groups did not show any significant difference regarding waist/hip ratio. While the body fat percentage was reduced in the mobile social networking group in the 4th, 8th, and 12th week of intervention, the differences between the two groups was not significant.

**Conclusion:**

Both text messaging and mobile social networking were effective in promoting physical activity and reducing anthropometric indices except for waist/hip ratio and body fat percentages, but the effect of mobile social networking was more pronounced. Thus, mobile social networking is recommended for promoting physical activity among middle-aged women.

## Background

Physical inactivity is a health issue worldwide, and according to the World Health Organization, 23% of adults and 81% of adolescents lack sufficient physical activity [1]. The prevalence of physical inactivity among the Iranian population is 30–70% according to gender and age group [2]. Physical activity is reported to decline over age [3]. Among different age groups, middle-aged women are more vulnerable to physical inactivity as one of the consequences of menopause. A Chinese study on 3920 participants found that 63.9% of middle-aged women and 61.95% of their male counterparts were physically inactive [4]. A study by Mohebi et al. showed that out of the 30,541 adults from 30 provinces in Iran, the prevalence of physical inactivity was 54.7% in a total population of whom 61.9% were adult women [5].

Physical inactivity increases the risk of chronic diseases such as cardiovascular disease, diabetes, obesity, cancer, and stroke [6]. Physical inactivity is responsible for a significant number of the non-communicable disease outbreaks and caused 15.2% of all deaths in the US in 2015 [7]. A large-scale study in Iran showed that important risk factors for morbidity and mortality among Iranian population aged 15–49 years were dietary risks, physical inactivity, high blood pressure, high body mass index, and smoking [8]. In addition, low physical activity has been reported to have a strong association with dementia [9].

An empowerment-focused intervention that is aimed to promote physical activity can be particularly successful among women [10]. A systematic review of 13 studies showed that SMS intervention has the potential to significantly promote physical activity [11]. Yamashita et al. [12] in their study on 39 older female adults with age more than 65 years found that social networking plus monthly incentives could significantly promote the level of physical activity among these women. Litwin and Shaul [13] in their study on 17,104 elderly individuals in Israel found that those with high social connectivity have vigorous physical activity. Also, a study by Nam et al. [14] on 64 female students with premenstrual syndrome (PMS) and physical inactivity showed that using social-media-based support through mobile applications could significantly reduce the PMS and promote physical activity compared to the control group. Other studies also evaluated the effect of text-messaging on physical activity. For example, Buchholz et al. in a systematic review including 10 studies found that sending text messages alone or text messaging combining with educational materials could significantly enhance physical activity [15]. Another study by Gell et al. on the effect of text messaging on the level of physical activity of working women showed that text messaging could increase self-efficacy as well as mean steps count per day [16].

To the best of our knowledge, no study has yet compared the effect of text messaging and that of with social networks on promoting physical activity in middle-aged women. The present study was performed to compare the effect of text messaging and that of mobile social networks on physical activity and anthropometric indices of middle-aged women in Iran.

## Methods

This was a randomized controlled trial (parallel design) in which 110 middle-aged women with physical inactivity were recruited and randomly assigned into two groups of text messaging and using social media to receive intervention for promoting physical activity. The inclusion criteria were age range of 40–60 years, basic digital literacy, having smart phone, access to the Internet, low score of International Physical Activity Questionnaire (IPAQ) or score of metabolic equivalent (MET) < 600 per week. Women with following criteria were excluded from the study: prohibition from physical activity for medical reasons including class 3 and 4 heart disease, and severe arthritis, receiving another training program for physical activity, membership in a gym, and pregnancy.

The design of this study was approved by the Ethics Committee of Ahvaz Jundishapur University of Medical Sciences (Ref. No: IR.AJUMS.REC.1396.727) and the protocol was registered in the Iranian Registry of Randomized Clinical Trials (IRCT) (Ref. No: IRCT20170916036223N1, 29/12/2017). All participants provided written informed consent before data collection. This study started in November 2018 and completed in March 2019.

### Sample size

The following formula was used for sample size calculation:

$$\begin{aligned} & {\text{n}} = \frac{{\left( {{\text{Z}}_{{{1} - \alpha /{2}}} + {\text{ Z}}_{{{1} - \beta }} } \right)^{{2}} \left( {{\text{S}}_{1}^{2} + {\text{S}}_{2}^{2} } \right)}}{{\left( {{\text{X}}_{{1}} {-}{\text{ X}}_{{2}} } \right)^{{2}} }} \\ & \begin{array}{*{20}c} {{\text{Z}}_{{{1} - \alpha /{2}}} = {1}/{96}} & {{\text{Z}}_{{{1} - \beta }} = {1}.{28}} & {{\text{S}}_{{1}} = {6}.{5}} & {{\text{S}}_{{2}} = {6}.{9}} \\ \end{array} \\ & \begin{array}{*{20}c} {{\text{X}}_{{1}} = {153}} & {{\text{X}}_{{2}} = {157}.{7}} & {n = \frac{{\left( {1.96 + 1.28} \right)2\left( {6.5 + 6.9} \right)2}}{{\left( {153 - 157.7} \right)2}} = 43} \\ \end{array} \\ \end{aligned}$$

We considered 25% for attrition and the total sample size was calculated to be 55.

### Recruitment

Middle aged women that had been registered in two centers (a Menopausal Counseling clinic and a public health center) in Ahvaz, Iran received a phone call requesting them to participating in the study. A total of 131 women were assessed according to the eligibility criteria, of whom 110 were eligible and provided their consent to participate.

### Randomization

A random table generated by EXCEL software was used for randomization (done by the study statistician). Due to the nature of this study, blinding of participants and researchers was not possible, but the code dedicated to each participant was kept with the secretary of the menopausal clinic, and neither the researcher nor the participants knew the code until the commencement of the trial.

### Measurements

A demographic questionnaire, a check list, and the International Physical Activity Questionnaire (IPAQ) were used for data collection. The demographic questionnaire included questions on age, age of menopause, level of schooling, marital status, occupation and number of children. The checklist was used to record anthropometric indices (height, weight, body mass index, waist circumference, hip circumference, waist/hip ratio, and percentage of body fat).

IPAQ contains 7 questions about physical activity over the past 7 days. It has three domains including physical activity related to work, leisure time, and home. In each domain, the number of days in which the person has been doing the activity for at least 10 min and the duration of physical activity in terms of day and minutes are asked. The intensity of total physical activity was calculated by means of metabolic equivalence (MET). Each MET represents the amount of energy consumed per minute for one person at rest. Each MTE is equivalent to 3.5 mm of oxygen consumed per one kilogram of body weight. If the total energy expended for vigorous physical activity is 1500 MET/min/week in at least 3 days from the last 7 days, or if the total energy expended for a combination of moderate and vigorous physical activity or walking is 3000 MET/min/week, the intensity of physical activity is considered intense. Having intense physical activity for 3 days or more, having 5 days of moderate physical activity, walking for at least 30 min a day, or having intense activity with total energy expenditure equaling to 600 MET/week were considered as moderate-intensity physical activity. If there is no physical activity or the activities do not meet the above conditions, they are considered low intensity [17]. The IPAQ was developed by an international group in Geneva in 1998. This questionnaire is adapted for the determination of physical activity of adults aged 15–69 and has been used in numerous studies in different countries. The validity and reliability of this questionnaire has been approved in Iran by Baghiani et al. [18].

To measure the participants' height and weight, two digital scales (Omron, China made) were used respectively. The weight of the participants was measured as they were barefoot in light clothing. Their height was measured with the Omron stadiometer, while they were standing barefoot. Body mass index (BMI) was calculated using a formula introduced by the World Health Organization (WHO) [19].

To measure the girth of the waist and hips, a flexible measuring tape was used [20]. The German Bauer diagnostic scale (BG64 USB) was used for measuring body fat percentage. After turning on the device, it was placed on a flat surface and the women were requested to step barefooted. Participants' characteristics, such as age, gender, weight, and level of physical activity were recorded in the device. The percentages of body fat, water, muscle, and bone mass, as well as basal metabolism were measured and recorded [21]. All measurements at baseline, 4, 8, and 12 weeks after intervention were done by a trained midwife.

### Intervention

The two groups were given instructions to promote their physical activity either through text messaging sent on mobile or mobile social networking. For the social networking group, a Telegram group was set up and all women (n = 55) were asked to join this group. Both groups received information about the need for physical activity, the benefits of physical activity, barriers to doing physical activity, and how to overcome these barriers. Women in the mobile text messaging group received text messages involving the same content as that presented to the mobile social networking group three times a week.

The women in two groups were requested to complete IPAQ prior to the intervention, as well as four, eight and twelve weeks after the intervention. Furthermore, anthropometric indices were measured over the aforementioned periods. All women kept journals to record their physical activity. Participants in both groups received training on how to record their physical activity for instance they should record those physical activities that lasted more than 10 min.

Upon completion of the study, individuals from both groups received payment for their Internet usage (CONSORT checklist, supplementary material 1).

### Statistics

Data analysis was performed using SPSS version 23. To compare the quantitative variables in the two groups, the independent *t*-test was used if the distribution was normal, and if it was not normal, the Mann–Whitney test was used, which was also used for qualitative variables. The Chi-square test was used for nominal or qualitative data. Paired *t*-test and Wilcoxon test were used for within group comparisons before and after the intervention. To compare the changes in each group in weeks 4, 8, and 12, the repeated measures test was used if the data were normal, and otherwise, the Friedman test was used. The significance level was set at < 0.05.

## Results

**Fig. 1:**
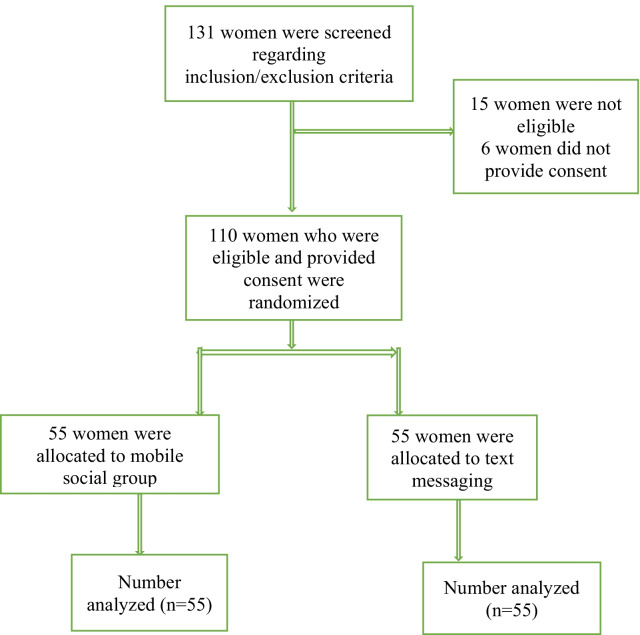
Flowchart of recruitment and retention of participants in the study

In this study 110 women were recruited, and there were not any dropouts (Fig. 1). The demographic characteristics of participants are listed in Table 1. The age of participants was 52.12 ± 6.07 and 51.18 ± 5.85 in the text messaging and mobile social networking groups respectively. The two groups did not show any significant difference regarding demographic characteristics.

**Table 1:** Demographic characteristics of participants in two groups of text message and mobile social networking

Variable	Text message (N = 55)	Mobile social networking (N = 55)	*P* value
	Mean ± SD		
Age (years)	52.12 ± 6.07	51.18 ± 5.85	0.409
*Marital status*			
Married	52 (94.5)	53 (96.4)	0.64
Single	3 (5.5)	2 (3.6)	
*Education*			
High school	9 (16.3)	7 (12.7)	0.89
Diploma	31 (56.3)	36 (65.4)	
University education	15 (26.2)	11 (21.8)	
*Menopausal status*			
Menopause	38 (69.1)	31 (56.4)	0.16
Pre-menopause	17 (30.9)	24 (43.6)	

Table 2 shows the physical activity of participants in two groups of text message and mobile social networking. As evident in this table, most of the participants were inactive before the intervention, while after the intervention, 81.8% and 14.5% of participants in the text message group had low and moderate physical activity. In the mobile social networking group, after 12 weeks, 23.6% and 72.7% of participants had low and moderate physical activity, respectively (*P* < 0.05).

**Table 2:** Physical activity of participants in two group of text message and mobile social networking

Groups’ Physical activity	Before intervention	4-weeks after intervention	8 weeks after intervention	12 weeks after intervention	*P* value using repeated measure test
	No (%)				
*Text message* (*N* = *55*)					
Inactive	25 (45.5)	18 (32.7)	7 (12.7)	2 (3.6)	< 0.001
Low	29 (52.7)	30 (54.5)	43 (78.2)	45 (81.8)	
Moderate	1 (1.8)	7 (12.7)	5 (9.1)	8 (14.5)	
*Mobile social networking* (*N* = *55*)					
Inactive	17 (30.9)	7 (12.7)	3 (5.5)	1 (1.8)	< 0.001
Low	38 (69.1)	34 (61.8)	22 (40)	13 (23.6)	
Moderate	0	14 (25.5)	30 (54.5)	40 (72.7)	
Vigorous	0	0	0	1 (1.8)	
*P* value	0.15	0.008	0.001	0.001	

Table 3 shows the mean physical activity in two groups of text message and mobile social networking. Before intervention, the two groups did not show any significant difference in terms of mean physical activity. Four weeks after the intervention, however, the mean of physical activity in text messaging group was higher than that in social networking (*P* = 0.008). The mean of physical activity in the mobile networking group compared to the text messaging group was significantly higher in the 8th week (2.89 ± 0.64 vs. 2.71 ± 0.49, *P* = 0.001) and 12th week of the intervention (3.30 ± 0.49 vs. 2.99 ± 0.49, *P* = 0.001) compared to the text messaging. The mean of energy expenditure in the 4th, 8th, and 12th weeks after intervention was also higher in the mobile social networking group in comparison to the text messaging group (*P* = 0.001).

**Table 3:** The mean intensity of physical activity in two groups of text message and mobile social networking

Variable	Text message (N = 55)	Mobile social networking (N = 55)	*P* value for *t*-test	*P* value for repeated measure
	Mean ± SD			
*Physical activity*				
Before intervention	1.92 ± 0.24	1.55 ± 0.01	0.07	< 0.001
4 weeks after intervention	2.38 ± 0.54	2.26 ± 0.57	0.008	
8 weeks after intervention	2.71 ± 0.49	2.89 ± 0.64	0.001	
12 weeks after intervention	2.99 ± 0.49	3.30 ± 0.49	0.001	
*P* value	0.001	0.001		
*Energy expenditure during past week*				
Before intervention	205.4 ± 374.4	81.3 ± 90.62	0.19	< 0.001
4 weeks after intervention	382.5 ± 475.4	762.7 ± 676.9	0.001	
8 weeks after intervention	475.7 ± 419.1	1196.5 ± 764.8	0.001	
12 weeks after intervention	555 ± 518.9	1631.3 ± 828	0.001	
*P* value	0.001	0.001		

The means of body weight and body mass index (BMI) before and after intervention are listed in Table 4. Participants of the two groups did not show any significant difference before the intervention, as well as in the 4th, and 8th weeks of intervention regarding weight and BMI. However, in the 12th week of intervention, participants in the mobile social networking group lost more weight and the reduction in their BMI was more pronounced compared to the text messaging group (*P* < 0.05).

**Table 4:** Mean weight and body mass index, 4, 8, and 12 weeks after intervention in two groups of text message and mobile social networking

Variable	Text message (N = 55)	Mobile social networking (N = 55)	*P* value	*P* value for repeated measure test
	Mean ± SD			
*Weight*				
Before intervention	79.16 ± 14.5	76.07 ± 10.08	0.19	0.08
4 weeks after intervention	78.6 ± 14.5	74.8 ± 10.3	0.17	
8 weeks after intervention	78.3 ± 14.4	73.9 ± 10.1	0.06	
12 weeks after intervention	77.9 ± 14.5	72.7 ± 10.03	0.03	
*P* value	0.02	0.001		
*Body mass index* (*kg/m*^2^)				
Before intervention	31 ± 5.5	29.9 ± 4	0.26	0.12
4 weeks after intervention	30.6 ± 5.23	29.5 ± 4.1	0.25	
8 weeks after intervention	30.6 ± 5.4	29.1 ± 4.06	0.09	
12 weeks after intervention	30.7 ± 5.8	28.6 ± 4.06	0.03	
*P* value	0.43	0.001		

Table 5 shows the anthropometric indices of the participants before and after the intervention. The waist circumference of women in the text messaging group before intervention was significantly more than that in the social networking group (*P* = 0.01). Four weeks after the intervention, the waist circumference of women in the text messaging reduced significantly compared with the mobile social networking group (*P* = 0.01), while in the 8th week of the intervention, the two groups did not show any significant difference in this regard. Twelve weeks after the intervention, the waist circumference of women in social networking group reduced significantly compared with the text messaging group (*P* = 0.001). The hip circumference of women in the mobile social networking group reduced significantly compared with the text messaging group 4, 8, and 12 weeks after intervention. Two groups did not show any significant difference regarding waist/hip ratio.

**Table 5:** Mean of anthropometric indices in two groups of text message and mobile social networking in 4, 8, and 12 weeks after intervention

Variable	Text messaging (N = 55)	Mobile social networking (N = 55)	*P* value	*P* value for repeated measure test
	Mean ± SD			
*Waist circumference *(*cm*)				
Before intervention	96.8 ± 12.01	90.8 ± 13.1	0.01	0.006
4 weeks after intervention	95.5 ± 11.7	90.6 ± 8.17	0.01	
8 weeks after intervention	93.6 ± 16.5	89.2 ± 8.7	0.08	
12 weeks after intervention	94.3 ± 11.8	87.8 ± 8.4	0.001	
*P* value	0.003	0.001		
*Hip circumference *(*cm*)				
Before intervention	111.7 ± 10.9	108.4 ± 8.9	0.09	0.01
4 weeks after intervention	109.5 ± 10.9	105 ± 8.01	0.01	
8 weeks after intervention	108.6 ± 11.2	103.2 ± 8.3	0.005	
12 weeks after intervention	107.7 ± 11.7	101.6 ± 8.1	0.002	
*P* value	0.001	0.001		
*Waist hip ratio*				
Before intervention	0.86 ± 0.06	0.85 ± 0.05	0.35	0.36
4 weeks after intervention	0.86 ± 0.06	0.85 ± 0.05	0.35	
8 weeks after intervention	0.87 ± 0.07	0.86 ± 0.06	0.3	
12 weeks after intervention	0.87 ± 0.06	0.86 ± 0.06	0.41	
*P* value	0.10	0.26		
*Body fat percentage*				
Before intervention	40.3 ± 5.6	40.3 ± 4.8	0.92	0.34
4 weeks after intervention	40.4 ± 5.4	39.5 ± 5.03	0.37	
8 weeks after intervention	40.2 ± 5.3	39.1 ± 4.9	0.28	
12 weeks after intervention	40.3 ± 5.1	38.5 ± 4.8	0.05	
*P* value	0.4	0.001		

While body fat percentage was reduced in the mobile social networking group in the 4th, 8th, and 12th weeks of intervention, but the differences between the two groups were not significant.

## Discussion

This study was designed to compare the effects of text messaging and mobile social networking on physical activity and anthropometric indices of middle-aged Iranian women. Our results showed that the frequency and the mean of physical activity significantly increased in the mobile social networking group compared to women in the text-messaging group. Also, the mean energy expenditure per week was significantly higher in the mobile social networking compared to the text messaging group.

In their study on 12 overweight or obese middle-aged women, Arigo et al. [22] found that connected networking and an automated physical activity sensor for four weeks could significantly promote the physical activity of these women. Peyman et al. [23] conducted a quasi-experimental study on 360 women and found that using media-based educational intervention for eight weeks could significantly increase the level of physical activity of women in the intervention group compared to the control group. The results of the present study are similar to those of Arigo et al. and Peyman et al. Although we found that using social networking was more effective for improving physical activity, but women in the text messaging also increased their physical activity with a lower speed. A systematic review included 12 studies by Alamnia et al. showed that using regular text messaging could improve the physical activity of people in developing countries [24].

Our results revealed there was a significant reduction in the weight and BMI of the participants in the mobile social networking group compared to the text messaging group. Similar to our results, Silina et al. in their study on 123 healthy overweight and obese individuals found that using text messaging could significantly reduce weight, body mass index, waist circumference, hip circumference, and fasting insulin after one year compared to the control group [25].

Our results revealed that waist and hip circumferences reduced significantly in the mobile social networking compared to the text messaging group. However, there were no significant differences between the groups in terms of waist/hip ratio and the body fat percentage. Increased waist circumference, BMI, and waist/hip ratio is a risk factor for coronary heart disease, and diabetes type 2 [26]. Sillina et al. found that weight, BMI, and WC decreased significantly in the intervention group who received instruction through text messaging in comparison to the control group after a one-year intervention [25], which is consistent with our results. Although there was a significant reduction in anthropometric indices such as weight, BMI, WC, and HC, the waist/hip ratio and body fat percentages did not decrease significantly, indicating that women need more than 12 weeks to achieve significant results regarding these two factors. In the present study, women in the social networking requested to join a Telegram group. Sometimes they had interaction with each other in the group and shared their experiences and this might affect the physical activity and anthropometric indices of participants beside of our intervention, while women in the text messaging group did not have any interaction with each other.

### Strengths and limitations of the study

This was the first study to compare the effects of text messaging and mobile social networking on improving physical activity and anthropometric indices among middle-aged women. However, it has certain limitations. First, we relied on responses of women about their physical activity level, which might be affected by recall bias. In this study, the participants and researchers were not blinded, and therefore the results may be affected by performance bias. For reducing this bias, all outcomes were measured by a single person who was not aware of the purpose of the study.

## Conclusion

This study provides evidence regarding intervention based on effectiveness of text messaging and mobile social networking in promoting physical activity and improving anthropometric indices in middle-age women, considering the effect of mobile social networking was more pronounced. The results of this study will help policymakers decide about the frequency of messages for health improvement of the population, especially middle-aged women.

## Data Availability

The datasets generated and/or analyzed during the current study are not publicly available due to restrictions (Ahvaz Jundishapur University of Medical Sciences does not permit to data publicity before publication) but are accessible through the corresponding author upon reasonable request.

## Abbreviations

PMS: Premenstrual syndrome
IPAQ: International Physical Activity Questionnaire
MET: Metabolic equivalent
BMI: Body mass index
WC: Waist circumference

## References

[1] World Health Organization. Physical Inactivity: a global public health problem. https://www.who.int/ncds/prevention/physical-activity/inactivity-global-health-problem/en/#:~:text=Globally%2C%2023%25%20of%20adults%20and,80%25%20in%20some%20adult%20subpopulations. Accessed 8 Apr 2021.

[2] Fakhrzadeh H, Djalalinia S, Mirarefin M, Arefirad T, Asayesh H, Safiri S, Samami E, Mansourian M, Qorbani M (2016). Prevalence of physical inactivity in Iran: a systematic review. J Cardiovasc Thorac Res.

[3] Ahmadi B, Amini Sanil N, Bani F, Bakhtari F (2018). predictors of physical activity in older adults in Northwest of Iran. Elder Health J.

[4] Zhou Y, Wu J, Zhang S, Yan S, He L, Mkandawire N (2018). Prevalence and risk factors of physical inactivity among middle-aged and older Chinese in Shenzhen: a cross-sectional study. BMJ Open.

[5] Mohebi F, Mohajer B, Yoosefi M, Sheidaei A, Zokaei H, Damerchilu B (2019). Physical activity profile of the Iranian population: STEPS survey, 2016. BMC Public Health.

[6] Anderson E, Durstine JL (2019). Physical activity, exercise, and chronic diseases: a brief review. Sports Med Health Sci.

[7] Carlson SA, Fulton JE, Pratt M, Yang Z, Adams EK (2015). Inadequate physical activity and health care expenditures in the United States. Prog Cardiovasc Dis.

[8] Forouzanfar MH, Sepanlou SG, Shahraz S, Dicker D, Naghavi P, Pourmalek F (2014). Evaluating causes of death and morbidity in Iran, global burden of diseases, injuries, and risk factors study 2010. Arch Iran Med.

[9] Kushkestani M, Enosrani S, Parvani M, Rezaei S (2020). The Relationship between the level of physical activity and dementia in elderly residents of nursing homes in Tehran. Biomed J Sci Tech.

[10] Segar M, Jayaratne T, Hanlon J, Richardson CR (2008). Fitting fitness into women's lives: effects of a gender-tailored physical activity intervention. Women Health Issues.

[11] Smith DM, Duque L, Huffman JC, Healy BC, Celano CM (2020). Text message intervention for physical activity: a systematic review and meta-analysis. Am J Prev Med.

[12] Yamashita R, Sato S, Akase R, Doi T, Tsuzuku S, Yokoi T (2021). Effects of social network incentives and financial incentives on physical activity and social capital among older women: a randomized controlled trial. BMC Public Health.

[13] Litwin P, Shaul A (2018). The effect of social network on the physical activity-cognitive function nexus in late life. Int Psychogeriatr.

[14] Nam SJ, Cha C (2019). Effects of social-media-based support on premenstrual syndrome and physical activity among female university students in South Korea. J Psychosom Obstet Gynecol.

[15] Buchholz SW, Wilbur J, Ingram D, Fogg L (2013). Physical activity text messaging interventions in adults: a systematic review. Worldviews Evid Based Nurs.

[16] Gell NM, Wadsworth DD (2013). The use of text messaging to promote physical activity in working women: a randomized controlled trial. J Phys Act Health.

[17] Zhang J, Brackbill D, Yang S, Becker J, Herbert N, Centola D (2016). Support or competition? How online social networks increase physical activity: a randomized controlled trial. Prev Med Rep.

[18] Baghiani Moghaddam MH, Bakhtari Aghdam F, Asghari Jafarabadi M, Allahverdipour H, Dabagh Nikookheslat S, Safarpour Sh (2012). The Iranian version of international physical activity questionnaire in Iran: content and construct validity, factor structure, internal consistency and stability. World Appl Sci.

[19] World Health Organization. Body mass index calculator. http://www.emro.who.int/nutrition/information-resources/bmi-calculator.html. Accessed 25 Oct 2021.

[20] World Health Organization. Waist circumference and waist–hip ratio: report of a WHO expert consultation, Geneva. 8–11 Dec 2008.

[21] Frey M. Body fat calculator: get an instant body fat percentage. https://www.verywellfit.com/how-to-use-body-fat-percentage-calculator-3858855. Accessed 25 Oct 2021.

[22] Arigo D, Schumacher LM, Pinkasavage E (2015). Addressing barriers to physical activity among women: a feasibility study using social networking-enabled technology. Ment Health Aware.

[23] Peyman N, Rezai-Rad M, Tehrani H, Gholian-Aval M, Vahedian-Shahroodi M, Miri HH (2018). Didital media-based health intervention on the promotion of women’s physical activity: a quasi-experimental study. BMC Public Health.

[24] Alamnia TT, Tesfaye W, Kelly M (2021). The effectiveness of text message delivered interventions for weight loss in developing countries: a systematic review and meta-analysis. Obes Rev.

[25] Silina V, Tessma MK, Senkane S, Krievina G, Bahs G (2017). Text messaging (SMS) as a tool to facilitate weight loss and prevent metabolic deterioration in clinically healthy overweight and obese subjects: a randomized controlled trial. Scand J Prim Health Care.

[26] Parker ED, Pereira MA, Stevens J, Folsom AR (2009). Association of hip circumference with incident diabetes and coronary heart disease: the atherosclerosis risk in communities study. Am J Epidemiol.

